# Assessment of the longitudinal changes in infarct heterogeneity post myocardial infarction

**DOI:** 10.1186/s12872-016-0373-5

**Published:** 2016-10-14

**Authors:** Idan Roifman, Nilesh R. Ghugre, Tasnim Vira, Mohammad I. Zia, Anna Zavodni, Mihaela Pop, Kim A. Connelly, Graham A. Wright

**Affiliations:** 1 Sunnybrook Research Institute, Schulich Heart Program, Sunnybrook Health Sciences Centre and the University of Toronto, 2075 Bayview Avenue, room M 315b, Toronto, ON M4N-3M5 Canada; 2 Keenan Research Centre for Biomedical Science and Li Ka Shing Knowledge Institute of St. Michael’s Hospital, The University of Toronto, Toronto, ON Canada; 3 Division of Cardiothoracic Imaging, Department of Medical Imaging, Sunnybrook Health Sciences Centre and the University of Toronto, Toronto, ON Canada; 4 Physical Sciences Platform, Sunnybrook Research Institute, Sunnybrook Health Sciences Centre and the University of Toronto, Toronto, ON Canada; 5 Department of Medical Biophysics, University of Toronto, Toronto, ON Canada

**Keywords:** ST elevation myocardial infarction (STEMI), Cardiac magnetic resonance imaging, Infarct heterogeneity, Gray zone/Peri-infarct zone

## Abstract

**Background:**

Infarct heterogeneity, as assessed by determination of the peri-infarct zone (PIZ) by cardiac magnetic resonance imaging, has been shown to be an independent predictor for the development of cardiac arrhythmias and mortality post myocardial infarction (MI). The temporal evolution of the PIZ post MI is currently unknown. Thus, the main objective of our study was to describe the temporal evolution of the PIZ over a 6 month time period in contemporarily managed ST elevation myocardial infarction (STEMI) patients. Further, given the poor prognosis associated with microvascular obstruction (MVO) post STEMI, we sought to compare the temporal evolution of the PIZ in patients with and without MVO. We hypothesized that patients with MVO would show a relative persistence of PIZ over time when compared to those without MVO.

**Methods:**

Twenty-one patients post primary percutaneous coronary intervention were enrolled and treated with evidence based therapy. Each patient had three cardiac MRI scans at 48 h, 3 weeks and 6 months post infarction. Repeated Measures Analysis of Variance (ANOVA) was used to assess the evolution of core infarct size and peri-infarct zone size across the three time frames.

**Results:**

The patients in this study were predominantly male, with ~40 % LAD territory infarction and a mean LVEF of 46 ± 7 %. Core infarct size and PIZ size both decreased significantly across the three time frames. The presence of microvascular obstruction (MVO), a known adverse prognostic factor, influenced PIZ size. Both patients with and without MVO had a significant reduction in core infarct size over time. Patients with MVO did not have a significant change in PIZ size over time (11.9 ± 6.8 %, 12.2 ± 7.5 %, 10.7 ± 6.6 % *p* = 0.77). In contrast, non-MVO patients did have a significant decrease in PIZ size over time (7.0 ± 5.5 %, 7.1 ± 6.5 %, 2.7 ± 2.6 %, *p* = 0.01).

**Conclusions:**

Peri-infarct zone size, like core infarct size, varies depending upon the timing of measurement. Patients with MVO displayed a persistence of the PIZ over time.

## Background

Cardiac magnetic resonance (CMR) imaging is considered the gold standard in the assessment of left ventricular structure and function [1, 2]. However, CMR’s major advantage over other imaging modalities lies in its ability to accurately detect altered tissue composition, referred to as tissue characterization. The ability of CMR to assess altered tissue composition has led to the identification of tissue signatures that predict adverse outcomes. One such measure is referred to as the peri-infarct zone (PIZ) or ‘gray zone’, which represents an area of infarct heterogeneity and has been shown to both correlate with cardiac arrhythmias and to be an independent predictor of post MI mortality [3–6]. Histological studies demonstrate that this zone consists of distorted bundles of surviving myocytes interweaved with extracellular matrix creating an arrhythmogenic substrate [7, 8]. The assessment of core infarct size (CIS) in the early period following myocardial infarction using late gadolinium enhancement (LGE) CMR may be misleading as the combination of necrosis/apoptosis and edema can lead to infarct size overestimation. As a result, it is suggested that LGE-CMR be performed greater than 48 h following acute myocardial infarction in order to accurately assess infarct size. Given that infarct size changes post myocardial infarction, in a temporal manner, we sought to compare the evolution of the heterogeneous peri-infarct zone in contemporarily managed ST elevation myocardial infarction (STEMI) patients across three time points – at 48 h post reperfusion, at 3 weeks and at 6 months following the index event, in order to better elucidate the variability of this measurement. Furthermore, multiple studies have linked the presence of MRI determined microvascular obstruction (MVO) with a poor clinical prognosis post STEMI [9–13]. We therefore sought to compare the temporal evolution of the PIZ in patients with and without MVO. We hypothesized that patients with MVO would show a relative persistence of PIZ over time when compared to those without MVO.

## Methods

### Study population

Twenty-one patients who presented to Sunnybrook Health Sciences Centre with STEMI were prospectively enrolled in our study between the years 2009–2011. Consent was obtained from all patients. The main inclusion criteria was patients who had met the diagnosis of STEMI. Exclusion criteria included failure to provide consent, significant arrhythmias, severe renal impairment (GFR <30 mL/min) as well as contraindications to CMR imaging such as implantable cardioverter-defibrillators and pacemakers. Six patients who had initially agreed to participate in the study dropped out (not included in the 21 patients mentioned above). Of these, 3/6 dropped out due to claustrophobia, two patients were lost to follow up and one decided not to continue with the study after his first scan. All patients underwent primary percutaneous coronary intervention (primary PCI) of the culprit coronary vessel. Further, all 21 patients received standard of care post STEMI medications including dual anti-platelet therapy, a statin and a beta-blocker. The study was approved by the ethics review board of Sunnybrook Health Sciences Centre.

### CMR acquisition protocol

Each patient was imaged at three time points: 48 h, 3 weeks, and 6 months post STEMI. Studies were performed on a 1.5-T clinical scanner (Signa Twinspeed HDx; GE Healthcare, Waukesha, WI) with image acquisition performed using electrocardiographic triggering and breath holds at end-expiration. Cardiac function was determined using contiguous short axis slices covering the left ventricle acquired with a standard SSFP (GE FIESTA) sequence with the following parameters: echo time = 1.6 ms, flip angle = 45°, acquisition matrix = 256 × 192, bandwidth = 125 kHz, and 20 cardiac phases per slice. An inversion recovery gradient echo T1-weighted sequence was used to assess for late gadolinium enhancement with the following parameters: TR = 5.5 msec, TE = 2.5 msec, FOV = 350 × 350 mm, matrix 192 × 128. The inversion time was manually adjusted to null signal from normal myocardium and short-axis images were obtained 10 min after intravenous administration of gadolinium-diethylene triamine pentaacetic acid (0.2 mmol/kg; Gadovist, Bayer Healthcare, Wayne, NJ) [14–17].

### CMR image analysis

Core infarct size, peri-infarct zone size and MVO were quantified from contrast-enhanced T1-weighted images utilizing the previously described full-width-half-maximum technique [14, 18, 19]. The algorithm was implemented using custom-written scripts developed in MATLAB (The Mathworks, Natick, MA). Specifically, the infarct core was defined as myocardium with signal intensity >50 % of the maximal myocardial signal intensity. A region of interest was then drawn in the remote myocardium (remote from the infarcted territory). Peri-infarct zone was subsequently defined as the myocardium with signal intensity > peak remote signal intensity but <50 % of the maximal signal intensity in the infarct core as described by Schmidt et al. [18]. MVO was identified as a region of hypoenhancement within the region of the hyperenhanced infarct on late gadolinium enhancement images and was manually traced. It was added to the core infarct size. Both core infarct size and peri-infarct zone were quantified as a percentage of LV mass.

T2 quantification was performed using a previously described free-breathing cardiac-gated spiral imaging sequence with T2 preparation. 5 short axis slices were equally spaced to cover the extent of the left ventricle from apex to base. The following parameters were utilized in this sequence; echo times = 2.9, 24.3, 88.2 and 184.2 milliseconds; 12 spiral interleaves with 4096 points each (16.4 millisecond duration) yielding a 1.4 mm in-plane resolution; slice thickness = 6 mm and bandwidth = 125kH [17, 20].

### Statistical analysis

Unpaired t-tests and chi-square tests were used to compare continuous and categorical variables between MVO and Non-MVO patients respectively. Results from these tests are displayed as means ± standard deviations (for t-tests) and as proportions (for chi-square tests). Repeated measures analysis of variance (ANOVA) was then used to compare the evolution of core infarct size and peri-infarct zone size across the three time frames. Tukey’s multiple comparison test was performed to compare preselected time frames (48 h vs. 3 weeks and 3 weeks vs. 6 months). MVO is a known predictor of adverse prognosis and infarct size. We therefore assessed the evolution of the peri-infarct zone, in patients with, and without MVO. In order to assess inter-observer variability in the assessment of the PIZ, a second experienced observer assessed one slice in 12 random patients (6 with MVO and 6 without) at the three time frames. This was done by calculating the Pearson correlation coefficient comparing the first and second observer. A Bland-Altman plot was also constructed comparing difference vs. average of observer 1 vs. observer 2. A p value <0.05 was considered statistically significant. Statistical analysis was done using GraphPad Prism 6.0 (GraphPad Software Inc., La Jolla, California).

## Results

**Fig. 1:**
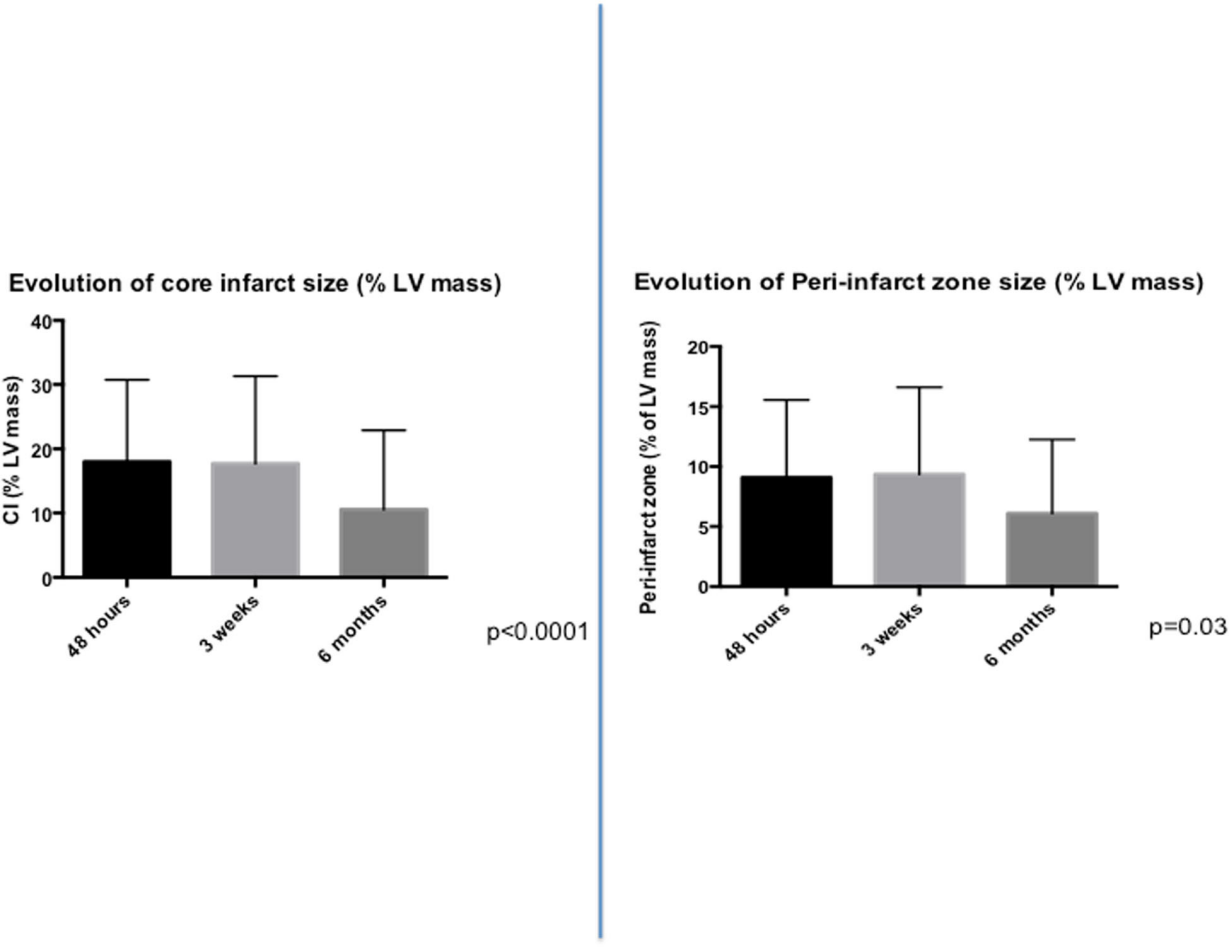
Evolution of core infarct size and peri-infarct zone (*gray zone*) in the entire patient population. Note, there is no difference between the 48 h and 3 week time point, whilst both reduce significantly by 6 months

Twenty-one patients were enrolled post STEMI. Table 1 displays the characteristics of the patient population. The mean age was 57 ± 10 years, 90 % of whom were male with a mean door to balloon time of 73 ± 25 min, and a peak CK of 2160 ± 1797 (IU). The mean LVEF was 46 ± 7 %. The infarcts were predominantly non-LAD territory. Approximately 38 % of the cohort was diabetic, 48 % was hypertensive, 38 % smoked and 19 % was dyslipidemic (see Table 1). Figure 1 demonstrates the effect of time upon core infarct and peri-infarct zone size in the entire cohort. As can be seen, both core infarct size (18.0 ± 12.7 %, 17.7 ± 13.6 %, 10.6 ± 12.4 %, *p* < 0.0001) and peri-infarct zone size (9.1 ± 6.5 %, 9.4 ± 7.2 %, 6.1 ± 6.1 %, *p* = 0.03) decreased significantly over the three time periods studied. Tukey’s multiple comparison test showed that in the case of both CIS and PIZ, there was no significant difference between the 48 h and 3 week time points. However, there was a significant difference between the 3 week and 6 month time points.

**Table 1:** Characteristics of the patient population

	Total (*N* = 21)
Age (years)	57 ± 10
Male (%)	19 (90)
Culprit vessel	
Left anterior descending	8 (38)
Circumflex	5 (24)
Right coronary artery	8 (38)
Multi-vessel coronary artery disease (%)	12 (57)
DM	8 (38)
Hypertension	10 (48)
Smoking	8 (38)
Dyslipidemia	4 (19)
Peak CK (AU)	2160 ± 1797
Door to Balloon time (mins)	73 ± 25
LVEF	46 ± 7
LVEDVi	66 ± 13
LVESVi	36 ± 9
RVEF	55 ± 12

Abbreviations used in the table

*DM* diabetes mellitus, *LVEF* left ventricular ejection fraction, *LVEDVi* (indexed left ventricular end diastolic volume), *LVESVi* (indexed left ventricular end systolic volume), *RVEF* right ventricular ejection fraction

**Fig. 2:**
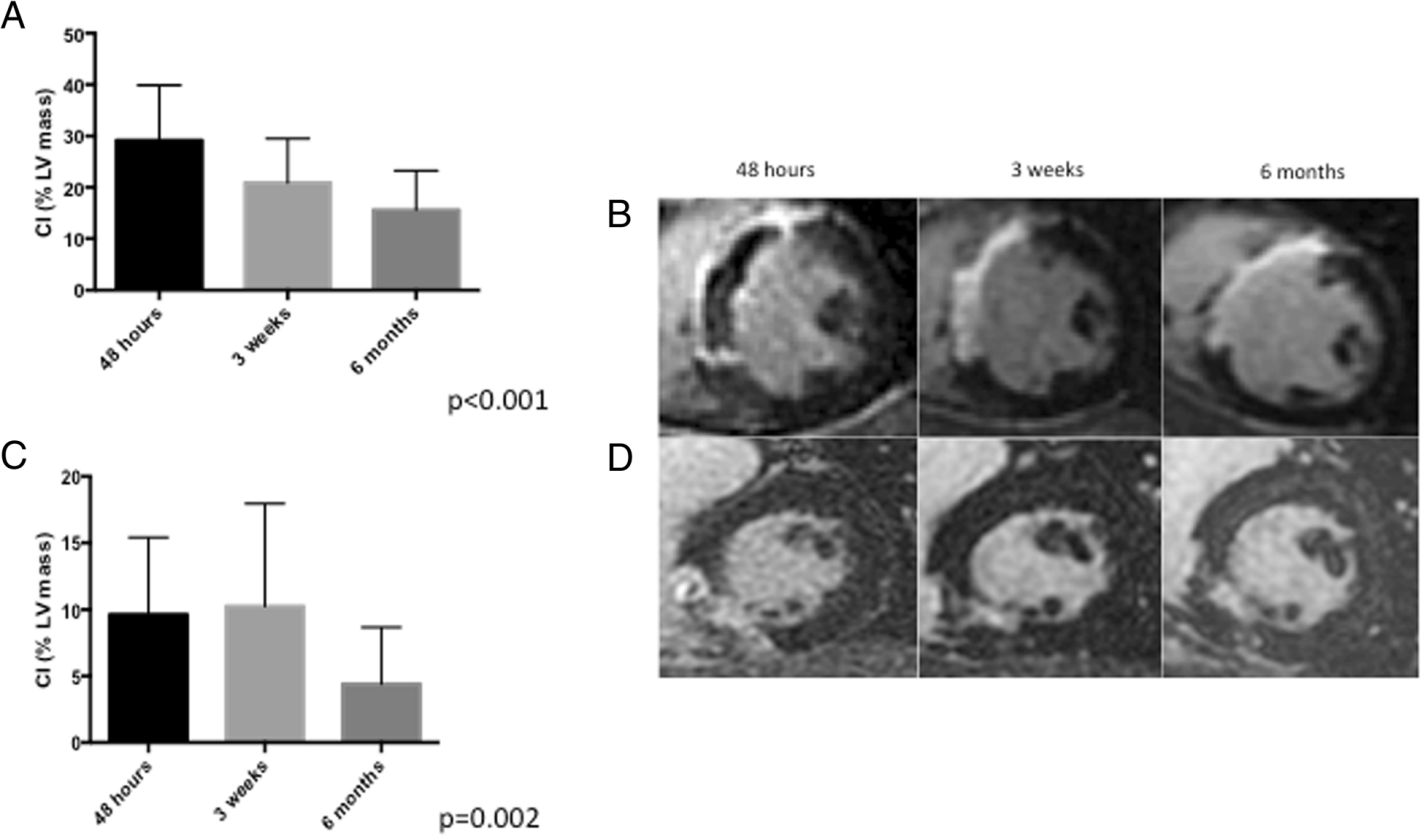
Evolution of core infarct size in patients with and without MVO. Panel (**a**) represents means and standard deviations of core infarct size, expressed as a percentage of LV mass, for all patients with MVO across the three time frames. Panel (**b**) represents the T1 weighted IR-GRE Late gadolinium enhancement images in a patient with MVO. Panel (**c**) represents means and standard deviations of core infarct size, expressed as a percentage of LV mass, for all patients without MVO across the three time frames. Panel (**d**) represents the T1 weighted IR-GRE Late gadolinium enhancement images in a patient without MVO

**Fig. 3:**
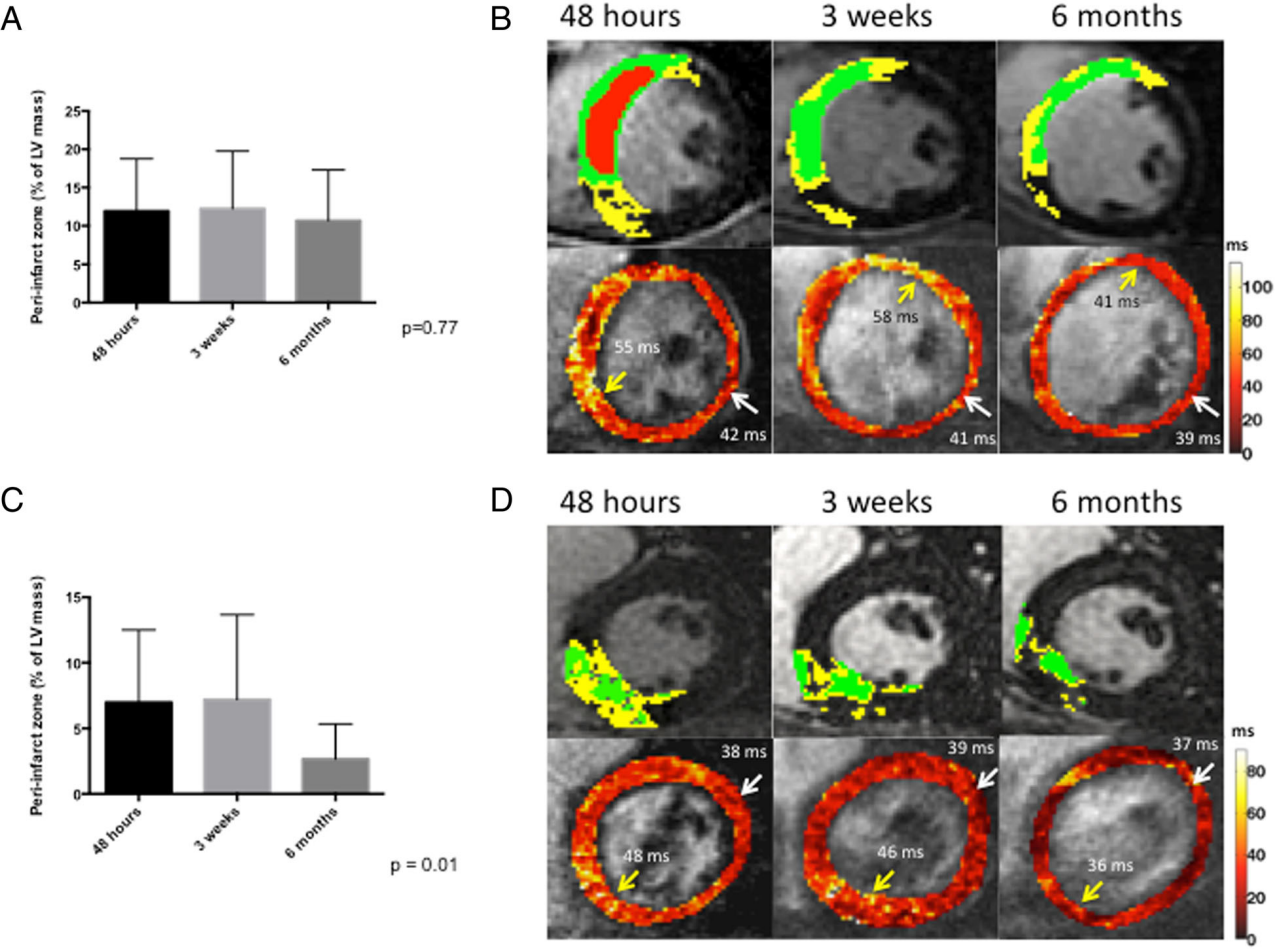
Evolution of the peri-infarct zone in patients with and without MVO. Panel (**a**) represents means and standard deviations of peri-infarct zone size, expressed as a percentage of LV mass, for all patients with MVO across the three time frames. Panel (**b**) represents T1 weighted IR-GRE Late gadolinium enhancement images in a patient with MVO with infarct core superimposed in *green* and PIZ superimposed in *yellow*. The *red* represents area of no reflow or MVO. Directly underneath, a panel displaying T2 maps at the three different time frames for the same patient is displayed. The *yellow* arrow indicates the T2 value in the PIZ and the *white* arrow indicates the T2 value in the remote myocardium. Panel (**c**) represents means and standard deviations of peri-infarct zone size, expressed as a percentage of LV mass, for all patients without MVO across the three time frames. Panel (**d**) represents the T1 weighted IR-GRE Late gadolinium enhancement images in a patient without MVO with infarct core superimposed in *green* and PIZ superimposed in *yellow*. Directly underneath, a panel displaying T2 maps at the three different time frames for the same patient is displayed. The *yellow* arrow indicates the T2 value in the PIZ and the *white* arrow indicates the T2 value in the remote myocardium. Of note, the patient with MVO has higher T2 values in the PIZ when compared to the patient without MVO at 48 h and 3 weeks but not at 6 months. Further, in both patients, T2 values in the PIZ are elevated at the first two time-frames when compared to the remote myocardium. At 6 months, T2 values are similar in the PIZ and the remote myocardium in both patients. A *yellow* patch is noted in the septal region in the patient without MVO at 6 months that is due to artifact from patient motion

Of the 21 patients in the overall study, nine had MVO and 12 did not. Patients with MVO had larger myocardial infarctions, as measured by greater elevation in peak creatine kinase (3209 ± 2123 vs. 1264 ± 906, *p* = 0.03) and a significantly reduced LVEF (41 ± 3 % vs. 49 ± 6,% *p* = 0.04) when compared to those without MVO. Otherwise, the two groups were similar with no significant differences in terms of mean age, gender, infarct territory, presence of multi-vessel coronary artery disease or rates of major cardiovascular risk factors (See Table 2). MVO patients also had larger core infarct sizes (as measured by LGE) when compared to Non-MVO patients across all three time points studied. Both MVO (29.2 ± 10.7 %, 21.0 ± 8.6 %, 15.6 ± 7.7 %, *p* < 0.001, see Fig. 2a) and non-MVO (9.6 ± 5.8 %, 10.3 ± 7.7 %, 4.4 ± 4.3 %, *p* = 0.002, see Fig. 2c) patients had a significant decrease in core infarct size over the three time frames. There was a significant reduction in peri-infarct zone size over time in the non-MVO patients (7.0 ± 5.5 %, 7.2 ± 6.5 %, 2.7 ± 2.6 %, *p* = 0.01, see Fig. 3c). In contrast, the peri-infarct zone was not significantly different in patients with MVO across the three time frames (11.9 ± 6.8 %, 12.2 ± 7.5 %, 10.7 ± 6.6 %, *p* = 0.77, see Fig. 3a).

**Table 2:** Characteristics of patients with and without microvascular obstruction (MVO)

	MVO *N* = 9	No MVO *N* = 12	*p*-value
Age (years)	56 ± 11	59 ± 10	0.66
Male (%)	6 (85)	11 (92)	0.83
Culprit vessel			
Left anterior descending	4 (44)	4 (33)	0.6
Circumflex	3 (33)	2 (17)	0.37
Right coronary artery	2 (22)	6 (50)	0.19
Multi-vessel coronary artery disease (%)	5 (56)	7 (58)	0.90
DM	3 (33)	5 (17)	0.7
Hypertension	4 (44)	6 (50)	0.8
Smoking	4 (44)	4 (33)	0.6
Dyslipidemia	1 (11)	3 (8)	0.42
Peak CK (AU)	3209 ± 2123	1264 ± 906	0.03
Door to Balloon time (mins)	78 ± 33	67 ± 20	0.5
LVEF	41 ± 3	49 ± 6	0.04
LVEDVi	67 ± 14	69 ± 10	0.7
LVESVi	39 ± 8	35 ± 8	0.55
RVEF	53 ± 7	55 ± 10	0.76

Abbreviations used in the table

*DM* diabetes mellitus, *LVEF* left ventricular ejection fraction, *LVEDVi* (indexed left ventricular end diastolic volume), *LVESVi* (indexed left ventricular end systolic volume), *RVEF* right ventricular ejection fraction

MVO patients had significantly elevated T2 values (in ms) in the peri-infarct zone when compared to the Non-MVO group at both 48 h (56.3 ± 6.8 vs. 48.4 ± 8.7, *p* = 0.04) and 3 weeks post infarct (56.5 ± 7.5 vs. 46.1 ± 7.5, *p* = 0.02). In contrast, there was no significant difference in T2 values in the peri-infarct zone of MVO vs. Non-MVO patients at the 6 month time frame (39.2 ± 4.7 vs. 38.0 ± 3.3, *p* = 0.53). When comparing T2 values in the peri-infarct zone to the remote myocardium, both MVO and Non-MVO patients showed a significant increase in T2 values at 48 h (MVO = 56.3 ± 6.8 vs. 42.7 ± 1.8, *p* <0.01, Non-MVO = 48.4 ± 8.7 vs. 39.2 ± 2.7, *P* < 0.01) and 3 weeks (MVO 56.5 ± 7.5 vs. 39.9 ± 2.4, *p* <0.01, Non-MVO = 46.1 ± 7.5 vs. 38.9 ± 2.1, *P* <0.01) but not at 6 months (MVO 39.2 ± 4.7 vs. 40.0 ± 1.1, *p* = 0.68 and Non-MVO = 38.0 ± 3.3 vs. 39.6 ± 1.7, *p* = 0.17).

**Fig. 4:**
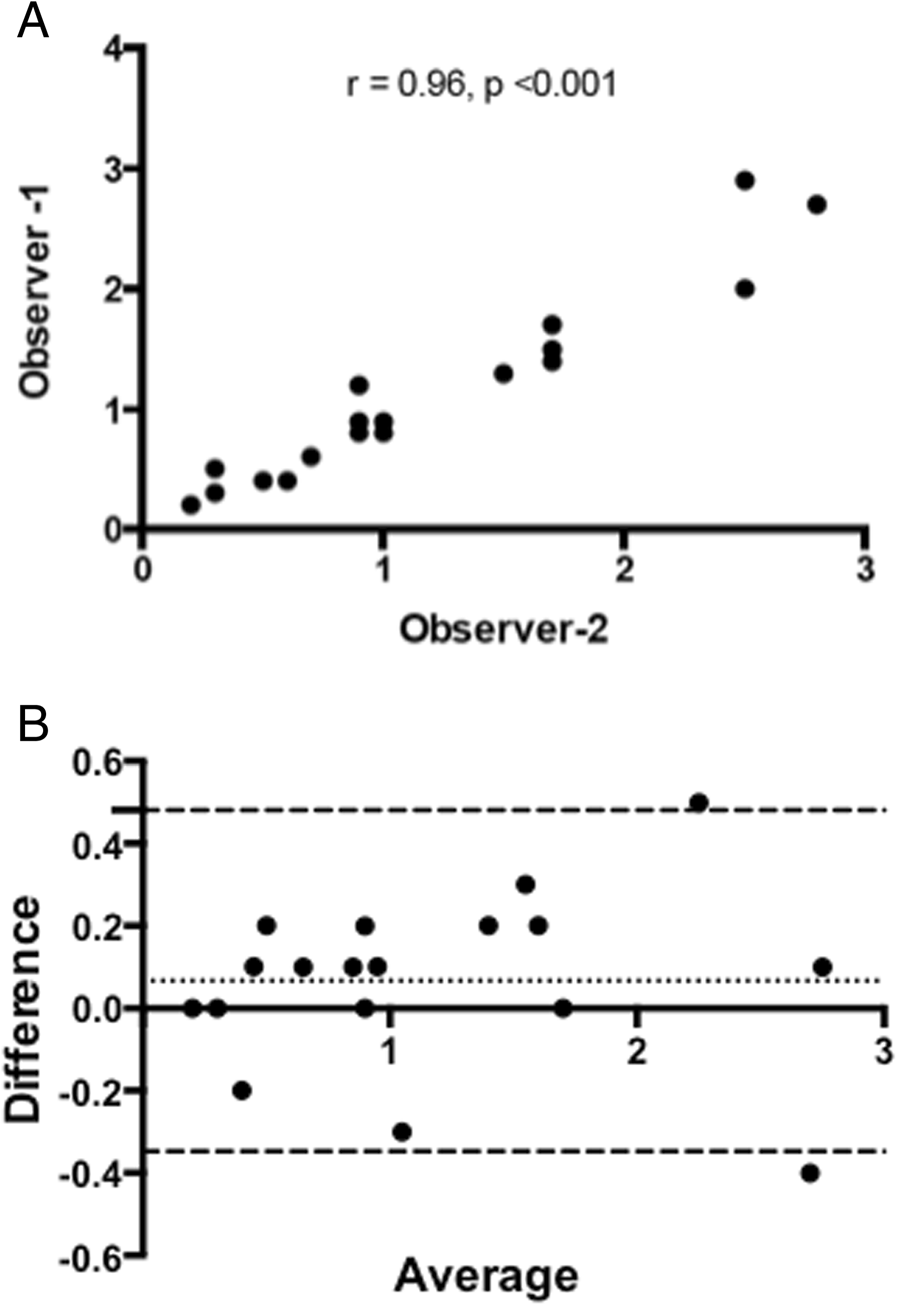
Inter-observer agreement in the assessment of peri-infarct zone. **a** Pearson correlation comparing observer 1 to observer 2. **b** Bland-Altman Plot assessing peri-infarct zone. Dashed lines represent the 95 % limits of agreement

Inter-observer agreement for the assessment of the PIZ was excellent (*r* = 0.96, *R* ^*2*^ = 0.93, *p* <0.001, see Fig. 4a). Bland-Altman plotting of difference vs. average of observer 1 vs. observer 2 resulted in the following: Bias = 0.07 and 95 % limits of agreement were between −0.35 and 0.48 (see Fig. 4).

## Discussion

Our study is the first to describe the evolution of the peri-infarct zone post STEMI. Our results demonstrate that peri-infarct zone, like core infarct size, varies significantly depending upon the time point at which it is measured post STEMI. We also demonstrate that MVO is associated with a relative persistence of the peri-infarct zone, whilst non-MVO patients demonstrate a significant reduction in the relative size of this zone over time.

Previous studies have shown that the peri-infarct border zone (‘gray zone’) is an important arrhythmogenic substrate. Roes et al., assessed 91 patients post myocardial infarction who were scheduled for ICD implantation for primary prevention purposes. They found that the extent of the peri-infarct zone was the only significant predictor of appropriate ICD therapy, both on univariate and multivariate analysis [21]. In another recently published study, Yang et al., showed that peri-infarct zone, as determined by multi-contrast late enhancement (MCLE) also predicts appropriate ICD therapy [22]. Yan et al. assessed peri-infarct zone in 144 patients who had late gadolinium enhancement on CMR in a pattern suggestive of previous myocardial infarction. Both STEMIs and NSTEMIs were included in this study. Their multivariate analysis found that the extent of the peri-infarct zone was an independent predictor for both cardiovascular and all-cause mortality in their population [3]. Our study adds to this body of literature as it demonstrates that the relative amount of peri-infarct zone varies over time post STEMI, and suggests that further studies assessing the peri-infarct zone should account for this temporal variability.

Microvascular obstruction is a known predictor of adverse outcomes, including cardiovascular death ost myocardial infarction. Cochet at al. assessed the prognostic significance of microvascular obstruction by cardiac magnetic resonance in 184 patients post acute myocardial infarction. CMR was performed within a week following the infarct. On univariate analysis, both core infarct size and MVO were associated with major adverse cardiac events (MACE). However, on multivariate analysis, only MVO was found to be an independent predictor of events [23]. The mechanism by which MVO leads to its poorer prognosis is currently unknown. In the era of contemporary cardiovascular care, including reperfusion therapy, cardiac arrhythmias remain an important cause of mortality post myocardial infarction [24]. Numerous studies have linked microvascular obstruction (MVO) with poor outcomes such as cardiac arrhythmias and death post myocardial infarction [4, 15, 25–29]. By demonstrating that patients with MVO do not see a reduction in peri-infarct zone over time post myocardial infarction, our findings provide further insight into how MVO confers poorer prognosis. Importantly, if these results are replicated in larger studies, the assessment of the peri-infarct zone may serve as a surrogate marker for risk, ultimately resulting in potential treatment implications.

The finding of increased myocardial T2, likely representing edema in the peri-infarct zone, is driven in part by a marked local inflammatory response. In keeping with this, we noted that at 6 months, during the chronic remodeling phase, T2 values in the peri-infarct zone normalize to values similar to those in the remote myocardium. Further, at 6 months, T2 values in the PIZ are similar between MVO and Non-MVO patients. Whilst the exact mechanism behind the poor prognosis observed with MVO remains unclear, we speculate that a marked early inflammatory response (as measured by T2 and edema) results in greater tissue injury and excessive replacement fibrosis. This then provides a substrate for arrhythmias and ongoing remodeling. However, this theory must be evaluated by future pre-clinical and large multi-center clinical studies to better assess mechanism and the link between evolution and size of the peri-infarct zone, MVO and downstream adverse clinical outcomes.

A recent systematic review and meta-analysis examining nine studies with a total of 4686 patients compared culprit only versus complete revascularization performed during the primary PCI procedure or during the index hospitalization in patients presenting with STEMI. The authors reported that there was no significant difference in major adverse cardiovascular events at 90 days and 1 year between the two strategies. However, there was a significant reduction in the rate of repeat revascularization at 1 year with a complete revascularization strategy [30]. In our study, a culprit only revascularization strategy was performed on the study patients. There was no significant difference in multi-vessel coronary artery disease between the MVO and non-MVO groups. Therefore, the differential effect that we reported in terms of PIZ evolution between the two groups cannot be explained by group differences in multi-vessel CAD or revascularization strategy.

There are a number of important limitations of this study. First, our study was a single center one that involved a small number of patients. Despite the small numbers, this is a unique cohort, which has undergone contemporary management and comprehensive CMR evaluation at multiple time frames, utilizing well validated CMR sequences on the same magnet, thus reducing systematic bias in the reporting of the results. Nonetheless, future larger studies will be needed in order to determine if the presence of MVO is an independent predictor for the evolution of the peri-infarct zone over time, after adjustment for other relevant clinical factors. Second, there is inherent variability in contouring the myocardium in order to calculate the PIZ. We therefore assessed inter-observer variability in the determination of the peri-infarct zone. This showed excellent inter-observer variability and was similar to results reported by other groups [31]. Third, determination of the size of the PIZ may be prone to partial volume averaging artifact from the infarct core [31]. Further, T2 values measured in the PIZ may also partially reflect this artifact. Future work using high resolution late gadolinium enhancement sequences may help address this issue.

## Conclusions

Both infarct size and peri-infarct zone (‘gray zone’) evolve over time following STEMI. In patients with MVO, there was a lack of regression of the peri-infarct zone.

## Abbreviations

AMI: Acute myocardial infarction
ANOVA: Analysis of variance
CIS: Core infarct size
CMR: Cardiac magnetic resonance
GZ: Gray zone
ICD: Implantable cardioverter defibrillator
MVO: Microvascular obstruction
PCI: Percutaneous coronary intervention
PIZ: Peri-infarct zone
STEMI: ST elevation myocardial Infarction

## References

[1] Schulz-Menger J, Bluemke DA, Bremerich J, Flamm SD, Fogel MA, Friedrich MG, Kim RJ, von Knobelsdorff-Brenkenhoff F, Kramer CM, Pennell DJ (2013). Standardized image interpretation and post processing in cardiovascular magnetic resonance: Society for Cardiovascular Magnetic Resonance (SCMR) board of trustees task force on standardized post processing. J Cardiovasc Magn Reson.

[2] Kawel-Boehm N, Maceira A, Valsangiacomo-Buechel ER, Vogel-Claussen J, Turkbey EB, Williams R, Plein S, Tee M, Eng J, Bluemke DA (2015). Normal values for cardiovascular magnetic resonance in adults and children. J Cardiovasc Magn Reson.

[3] Yan AT, Shayne AJ, Brown KA, Gupta SN, Chan CW, Luu TM, Di Carli MF, Reynolds HG, Stevenson WG, Kwong RY (2006). Characterization of the peri-infarct zone by contrast-enhanced cardiac magnetic resonance imaging is a powerful predictor of post-myocardial infarction mortality. Circulation.

[4] Yan AT, Gibson CM, Larose E, Anavekar NS, Tsang S, Solomon SD, Reynolds G, Kwong RY (2006). Characterization of microvascular dysfunction after acute myocardial infarction by cardiovascular magnetic resonance first-pass perfusion and late gadolinium enhancement imaging. J Cardiovasc Magn Reson.

[5] Judd RM, Kim RJ (2006). Can cardiac MRI predict outcome in patients at risk for unrecognized myocardial infarction?. Nat Clin Pract Cardiovasc Med.

[6] Klocke FJ, Wu E, Lee DC (2006). “Shades of gray” in cardiac magnetic resonance images of infarcted myocardium: can they tell us what we’d like them to?. Circulation.

[7] Yao JA, Hussain W, Patel P, Peters NS, Boyden PA, Wit AL (2003). Remodeling of gap junctional channel function in epicardial border zone of healing canine infarcts. Circ Res.

[8] Ursell PC, Gardner PI, Albala A, Fenoglio JJ, Wit AL (1985). Structural and electrophysiological changes in the epicardial border zone of canine myocardial infarcts during infarct healing. Circ Res.

[9] Bodi V, Sanchis J, Nunez J, Mainar L, Lopez-Lereu MP, Monmeneu JV, Rumiz E, Chaustre F, Trapero I, Husser O (2009). Prognostic value of a comprehensive cardiac magnetic resonance assessment soon after a first ST-segment elevation myocardial infarction. J Am Coll Cardiol Img.

[10] Bodi V, Rumiz E, Merlos P, Nunez J, Lopez-Lereu MP, Monmeneu JV, Chaustre F, Moratal D, Trapero I, Blasco ML (2011). One-week and 6-month cardiovascular magnetic resonance outcome of the pharmacoinvasive strategy and primary angioplasty for the reperfusion of ST-segment elevation myocardial infarction. Rev Esp Cardiol.

[11] Bruder O, Breuckmann F, Jensen C, Jochims M, Naber CK, Barkhausen J, Erbel R, Sabin GV (2008). Prognostic impact of contrast-enhanced CMR early after acute ST segment elevation myocardial infarction (STEMI) in a regional STEMI network: results of the “Herzinfarktverbund Essen”. Herz.

[12] Choi CJ, Haji-Momenian S, Dimaria JM, Epstein FH, Bove CM, Rogers WJ, Kramer CM (2004). Infarct involution and improved function during healing of acute myocardial infarction: the role of microvascular obstruction. J Cardiovasc Magn Reson.

[13] Choi JW, Gibson CM, Murphy SA, Davidson CJ, Kim RJ, Ricciardi MJ (2004). Myonecrosis following stent placement: association between impaired TIMI myocardial perfusion grade and MRI visualization of microinfarction. Catheter Cardiovasc Interv.

[14] Zia MI, Ghugre NR, Connelly KA, Strauss BH, Sparkes JD, Dick AJ, Wright GA (2012). Characterizing myocardial edema and hemorrhage using quantitative T2 and T2* mapping at multiple time intervals post ST-segment elevation myocardial infarction. Circ Cardiovasc Imaging.

[15] Zia MI, Ghugre NR, Connelly KA, Joshi SB, Strauss BH, Cohen EA, Wright GA, Dick AJ (2012). Thrombus aspiration during primary percutaneous coronary intervention is associated with reduced myocardial edema, hemorrhage, microvascular obstruction and left ventricular remodeling. J Cardiovasc Magn Reson.

[16] Roifman I, Ghugre N, Zia MI, Farkouh ME, Zavodni A, Wright GA, Connelly KA (2016). Diabetes is an independent predictor of right ventricular dysfunction post ST-elevation myocardial infarction. Cardiovasc Diabetol.

[17] Roifman I, Zia MI, Zavodni A, Wolff R, Ghugre NR, Leber AW, Dick AJ, Wright GA, Connelly KA (2014). Evolution of right ventricular function post-acute ST elevation myocardial infarction. J Magn Reson Imaging.

[18] Schmidt A, Azevedo CF, Cheng A, Gupta SN, Bluemke DA, Foo TK, Gerstenblith G, Weiss RG, Marban E, Tomaselli GF (2007). Infarct tissue heterogeneity by magnetic resonance imaging identifies enhanced cardiac arrhythmia susceptibility in patients with left ventricular dysfunction. Circulation.

[19] Detsky JS, Paul G, Dick AJ, Wright GA (2009). Reproducible classification of infarct heterogeneity using fuzzy clustering on multicontrast delayed enhancement magnetic resonance images. IEEE Trans Med Imaging.

[20] Zia MI, Ghugre NR, Roifman I, Strauss BH, Walcarius R, Mohammed M, Sparkes JD, Dick AJ, Wright GA, Connelly KA (2014). Comparison of the frequencies of myocardial edema determined by cardiac magnetic resonance in diabetic versus nondiabetic patients having percutaneous coronary intervention for ST elevation myocardial infarction. Am J Cardiol.

[21] Roes SD, Borleffs CJ, van der Geest RJ, Westenberg JJ, Marsan NA, Kaandorp TA, Reiber JH, Zeppenfeld K, Lamb HJ, de Roos A (2009). Infarct tissue heterogeneity assessed with contrast-enhanced MRI predicts spontaneous ventricular arrhythmia in patients with ischemic cardiomyopathy and implantable cardioverter-defibrillator. Circ Cardiovasc Imaging.

[22] Yang Y, Connelly KA, Zeidan-Shwiri T, Lu Y, Paul G, Roifman I, Zia MI, Graham JJ, Dick AJ, Crystal E, Wright GA (2013). Multi-contrast late enhancement CMR determined gray zone and papillary muscle involvement predict appropriate ICD therapy in patients with ischemic heart disease. J Cardiovasc Magn Reson.

[23] Cochet AA, Lorgis L, Lalande A, Zeller M, Beer JC, Walker PM, Touzery C, Wolf JE, Brunotte F, Cottin Y (2009). Major prognostic impact of persistent microvascular obstruction as assessed by contrast-enhanced cardiac magnetic resonance in reperfused acute myocardial infarction. Eur Radiol.

[24] Solomon SD, Zelenkofske S, McMurray JJ, Finn PV, Velazquez E, Ertl G, Harsanyi A, Rouleau JL, Maggioni A, Kober L (2005). Sudden death in patients with myocardial infarction and left ventricular dysfunction, heart failure, or both. N Engl J Med.

[25] Klem I, Kim RJ (2008). Assessment of microvascular injury after acute myocardial infarction: importance of the area at risk. Nat Clin Pract Cardiovasc Med.

[26] de Waha S, Desch S, Eitel I, Fuernau G, Lurz P, Leuschner A, Grothoff M, Gutberlet M, Schuler G, Thiele H (2012). Relationship and prognostic value of microvascular obstruction and infarct size in ST-elevation myocardial infarction as visualized by magnetic resonance imaging. Clin Res Cardiol.

[27] Klug G, Mayr A, Schenk S, Esterhammer R, Schocke M, Nocker M, Jaschke W, Pachinger O, Metzler B (2012). Prognostic value at 5 years of microvascular obstruction after acute myocardial infarction assessed by cardiovascular magnetic resonance. J Cardiovasc Magn Reson.

[28] Malek LA, Spiewak M, Klopotowski M, Misko J, Ruzyllo W, Witkowski A (2012). The size does not matter - the presence of microvascular obstruction but not its extent corresponds to larger infarct size in reperfused STEMI. Eur J Radiol.

[29] Wong DT, Richardson JD, Puri R, Nelson AJ, Bertaso AG, Teo KS, Worthley MI, Worthley SG (2012). The role of cardiac magnetic resonance imaging following acute myocardial infarction. Eur Radiol.

[30] Moretti C, D’Ascenzo F, Quadri G, Omede P, Montefusco A, Taha S, Cerrato E, Colaci C, Chen SL, Biondi-Zoccai G, Gaita F (2015). Management of multivessel coronary disease in STEMI patients: a systematic review and meta-analysis. Int J Cardiol.

[31] Schelbert EB, Hsu LY, Anderson SA, Mohanty BD, Karim SM, Kellman P, Aletras AH, Arai AE (2010). Late gadolinium-enhancement cardiac magnetic resonance identifies postinfarction myocardial fibrosis and the border zone at the near cellular level in ex vivo rat heart. Circ Cardiovasc Imaging.

